# Multi-omic assessment shows dysregulation of pulmonary and systemic immunity to e-cigarette exposure

**DOI:** 10.1186/s12931-023-02441-2

**Published:** 2023-05-25

**Authors:** David P. Scieszka, Devon Garland, Russell Hunter, Guy Herbert, Selita Lucas, Yan Jin, Haiwei Gu, Matthew J. Campen, Judy L. Cannon

**Affiliations:** 1 Department of Pharmaceutical Sciences, University of New Mexico School of Pharmacy, University of New Mexico Health Sciences Center, Albuquerque, NM USA; 2 Department of Molecular Genetics and Microbiology, University of New Mexico School of Medicine, MSC 08 4660, 1 University of New Mexico, Albuquerque, NM 87131 USA; 3 Center for Translational Science, Florida International University, Port St. Lucie, FL USA; 4 Autophagy, Inflammation, and Metabolism Center of Biomedical Research Excellence, University of New Mexico School of Medicine, Albuquerque, NM USA

**Keywords:** E-cigarettes, Lung immunity, T cells, Immune modulation, Metabolomics, Lipidomics, Multi-omic analyses

## Abstract

Electronic cigarette (Ecig) use has become more common, gaining increasing acceptance as a safer alternative to tobacco smoking. However, the 2019 outbreak of Ecig and Vaping-Associated Lung Injury (EVALI) alerted the community to the potential for incorporation of deleterious ingredients such as vitamin E acetate into products without adequate safety testing. Understanding Ecig induced molecular changes in the lung and systemically can provide a path to safety assessment and protect consumers from unsafe formulations. While vitamin E acetate has been largely removed from commercial and illicit products, many Ecig products contain additives that remain largely uncharacterized. In this study, we determined the lung-specific effects as well as systemic immune effects in response to exposure to a common Ecig base, propylene glycol and vegetable glycerin (PGVG), with and without a 1% addition of phytol, a diterpene alcohol that has been found in commercial products. We exposed animals to PGVG with and without phytol and assessed metabolite, lipid, and transcriptional markers in the lung. We found both lung-specific as well as systemic effects in immune parameters, metabolites, and lipids. Phytol drove modest changes in lung function and increased splenic CD4 T cell populations. We also conducted multi-omic data integration to better understand early complex pulmonary responses, highlighting a central enhancement of acetylcholine responses and downregulation of palmitic acid connected with conventional flow cytometric assessments of lung, systemic inflammation, and pulmonary function. Our results demonstrate that Ecig exposure not only leads to changes in pulmonary function but also affects systemic immune and metabolic parameters.

**Supplementary Information:**

The online version contains supplementary material available at 10.1186/s12931-023-02441-2.

## Introduction

The use of electronic nicotine delivery systems (ENDS, also called e-cigarettes or vaping) has surged in recent years, with over 10 million adults and 3 million adolescents actively using ENDS [1]. The FDA commissioner has used the term “epidemic” levels to describe the ubiquity of vaping. However, a recent spate of hospitalizations and deaths in 2019 due to Ecig or vaping-associated acute lung injury (EVALI), as well as combined use with traditional cigarettes, suggests that ENDS may pose unanticipated risks to lung health [2]. While new products and components are being incorporated into vaping products, very little is known about specific effects of vaping on lung function, particularly impacts to immune function.

It is well known that inhaled toxicants including traditional cigarette smoking have significant impacts on immunity, including damage to respiratory epithelium [3, 4], as well as increased chronic inflammation with increased susceptibility to viral and bacterial respiratory infections [5]. Vaping has been associated with increased risk of COVID-19 [6–8], and experimental evidence shows that vaping in animal models increases vulnerability to influenza A virus (IAV) [9], and predisposes airway epithelial cells to bacterial infection [10]. However, the precise effects of vaping and ENDS liquids on immune cells and immune function remain understudied.

ENDS products predominately use a base carrier propylene glycol (PG) plus vegetable glycerin (VG) to act as one of the most common carriers for vaping liquids [11–14]. PG and VG are also widely used in the food and cosmetics industries, and effects of PG and/or VG on multiple biological systems including renal and respiratory function have been documented [15–21]. PGVG is used in combination with cutting agents such as Vitamin E acetate (VEA), which has been identified as the likely causal driver of EVALI, and thus its use mostly discontinued [1, 2, 22, 23]. Alternatives to VEA include Phytol which is a diterpene alcohol that may naturally exist in cannabis products in small amounts, but it was an inexpensive diluent that many manufacturers of cannabis products were considering incorporating into products as a principal ingredient and was found in counterfeit cartridges [24]. An initial study highlighted significant pulmonary toxicity from phytol in rats and, for the most part, this finding quickly circulated to manufacturers who removed phytol from products [25]. That study used a high concentration incorporation of phytol and observed overt morbidity and mortality. In the instance of EVALI, vitamin E acetate has similarly been shown to induce severe lung inflammation in rodent models [22, 26]. While these products have been appropriately removed from commercial products, it remains unclear how these chemicals promote lung injury and, thus, what other similar chemicals may also be unsafe for general inhalation. Agents continuously change, but PGVG remains more consistent, and a better understanding of potential effects of base vaping compounds is needed.

In this study, we developed an exposure system to expose animals to PGVG vapor to study both system as well as lung specific effects of vaping exposure. Using a combination of transcriptome, proteome, metabolome, lipidome, as well as flow cytometry analyses of immune cell populations, we aim to identify novel pathways that are impacted by vaping exposure to PGVG as well as the addition of phytol. We find that these vaping exposures have both lung-specific as well as systemic effects, suggesting that vaping may have broad consequences for immune function.

## Results

### Body weight and pulmonary function

We developed a customized, flexible exposure system using a Smok® G-Priv3 Mod, operated at 50W with a 0.17Ω nickel coil, with laboratory-prepared e-liquids of propylene glycol/vegetable glycerin (PGVG) (50% + 50%) with and without 1% phytol by volume. The exposure chamber was monitored in real-time for mass concentration (DustTrak II, TSI), targeting an average of 125 mg/m^3^ (Additional file 1: Fig. S1), controlled as a stable concentration from day to day, with aerosol size distribution (mmad typically 120-250 nm) for all exposures (for details of exposure system see Materials and Methods).

**Fig. 1:**
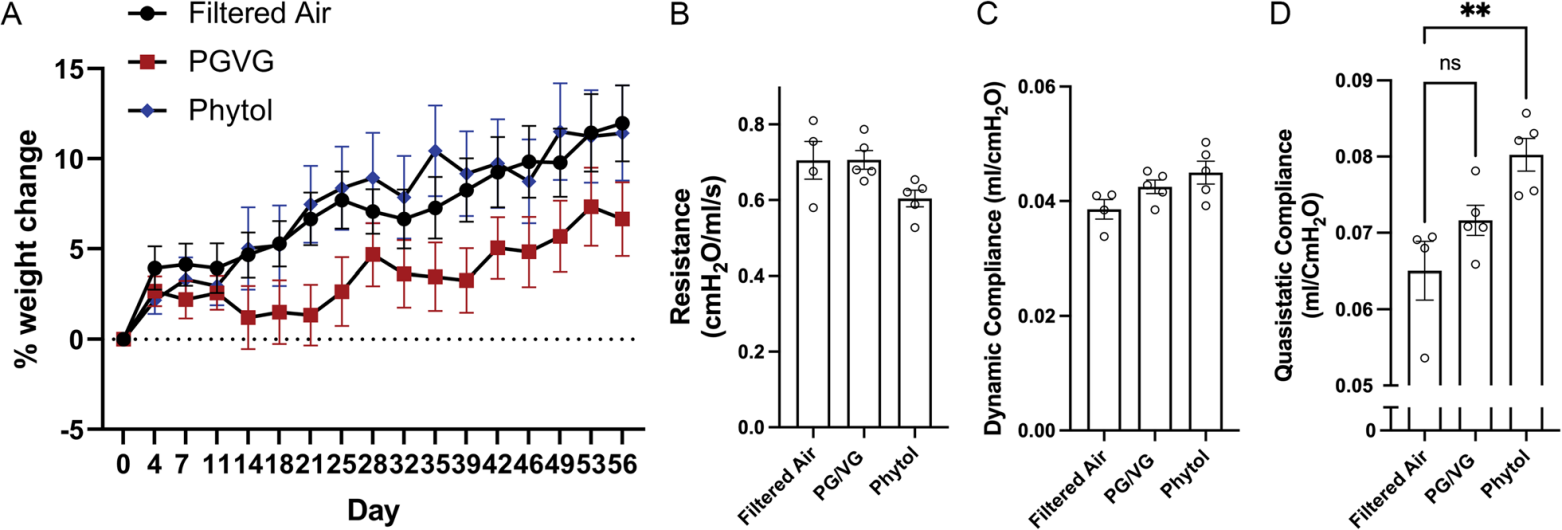
Changes to weight & respiratory function after vaping exposure. **A** Female mice 8–20 weeks were exposed to filtered air (control; FA), propylene glycol/vegetable glycerin (PGVG; 50/50) or PGVG + 1% phytol (Phytol) for 8 weeks, 2 h per day, 5 days per week. A) Animals were weighed every 3–4 days. N = 20/group. PGVG-exposed animals were significantly different from FA- and Phytol-exposed animals by 2-way ANOVA p < 0.0001. At terminus of the study, pulmonary function was analyzed using the Flexivent system for **B** resistance, **C** dynamic compliance, and **D** quasistatic lung compliance. ** indicates p < 0.01 by Students T-test between the 2 indicated conditions

**Fig. 2:**
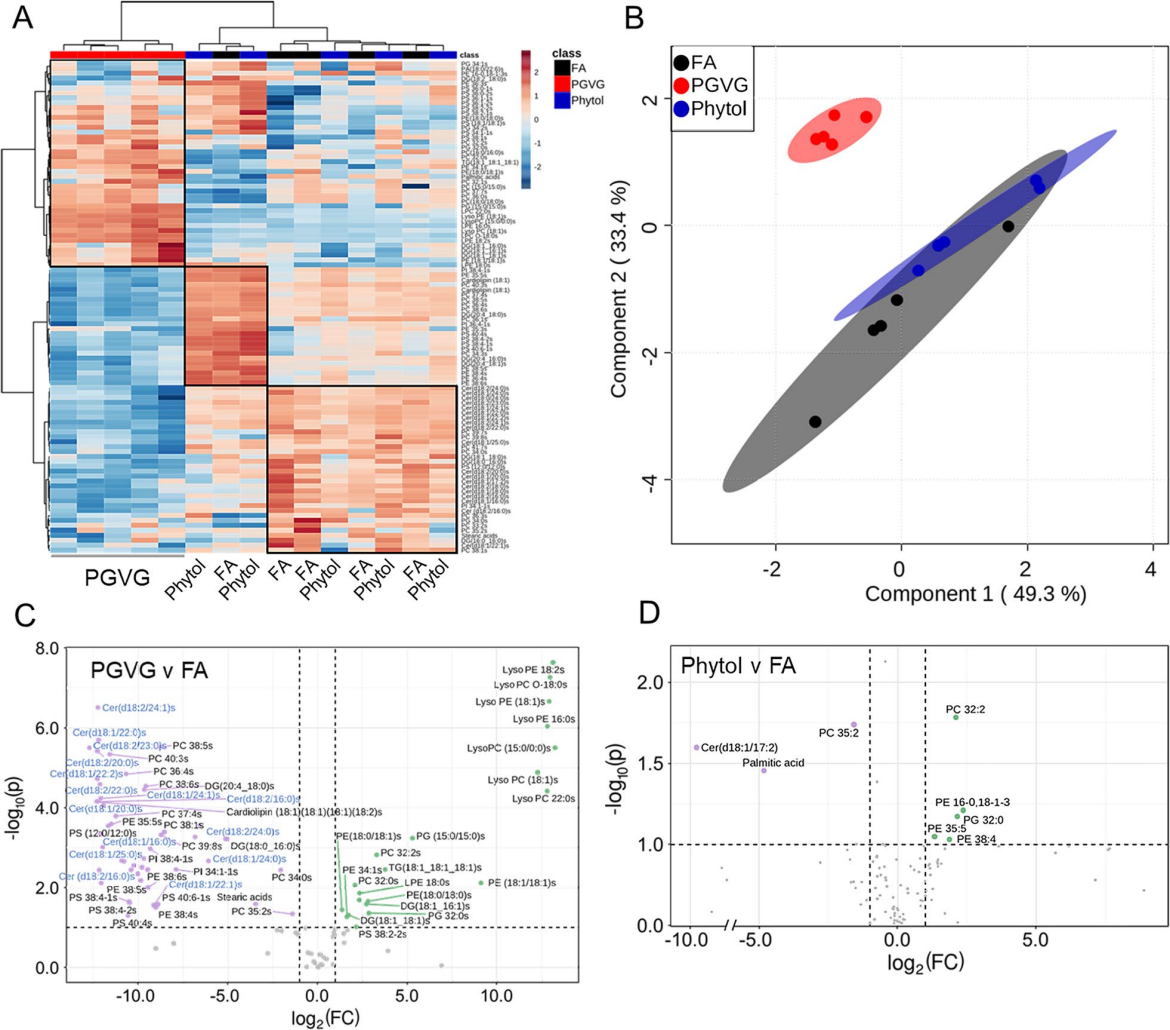
Alterations in lipids in lungs following exposure to Ecig components PGVG and Phytol. Lungs from individual animals from each group (n = 5/gp) were frozen and lipid extracted and analyzed for lipids as detailed in Materials and Methods. **A** Heat map comparing individual samples from the top 100 lipids; **B** Overlay of statistically significant lipid species using the partial least squares discriminant analysis (PLS-DA). **C**, **D** Volcano plot showing a comparison of statistically significantly differential lipids from lung of PGVG exposed **C** or Phytol exposed **D** versus filtered air (FA) control animals. Green indicates metabolites upregulated in PGVG **C** or Phytol **D** exposed lung relative to control. Purple indicates metabolites downregulated in PGVG **C** or Phytol **D** relative to control. For the full list of lipids differentially detected in Phytol versus FA and PGVG versus FA, see Table 1

C57BL/6 mice were exposed by inhalation for 2 h/day, 5 days/week × 8 weeks in order to model chronic Ecig use, similar to exposure times used in previous studies of chronic exposures between 4 and16 weeks [9, 27, 28]. We tracked weight change over the 8-week period and found that while all mice gained weight over the exposure period, animals exposed to PGVG gained significantly less weight than control Filtered Air (FA) exposed or phytol exposed animals (Fig. 1A). We then tested overall lung and respiratory function using the Flexivent system at the end of the 8-week exposure period. PGVG exposure did not significantly change overall respiratory resistance (Fig. 1B), dynamic compliance (Fig. 1C), or quasistatic compliance (Fig. 1D). In contrast, PGVG + phytol exposure significantly increased quasistatic compliance (Fig. 2C; p = 0.0036 compared to Filtered Air by ANOVA). PGVG + phytol trended but did not significantly affect airways resistance (Fig. 1B; p = 0.09 compared to Filtered Air) or dynamic compliance (Fig. 1C; p = 0.055 compared to Filtered Air). We also assessed systemic cardiovascular effects by ultrasonography, including heart rate, election fraction, stroke volume, and blood pressure, all of which showed no change as a result of any exposure (Additional file 2: Fig. S2). These results suggest that Ecig exposure can have specific and differential effects on metabolism and lung function.

### Lung lipidomics

**Fig. 3:**
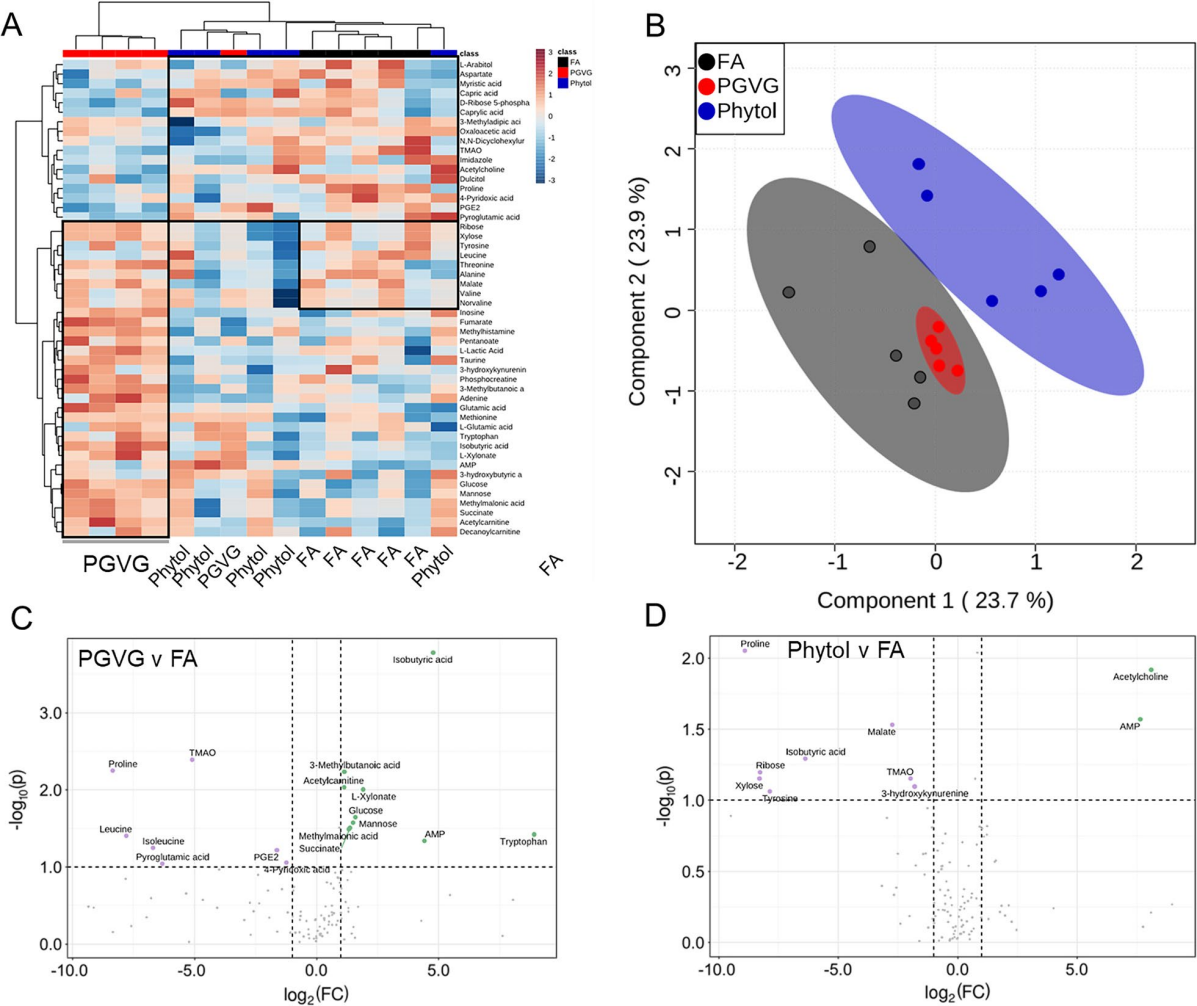
Alterations in metabolites in lungs following exposure to Ecig components PGVG and Phytol. Lungs from individual animals from each group (n = 5/gp) were frozen and metabolites extracted and analyzed for metaboites as detailed in Materials and Methods. **A** Heat map comparing individual samples from the top 50 metabolites; **B** Overlay of statistically significant metabolic species using the partial least squares discriminant analysis (PLS-DA). **C**, **D** Volcano plot showing a comparison of statistically significantly differential metabolites from lung of PGVG exposed **C** or Phytol exposed **D** versus filtered air (FA) control animals. Green indicates metabolites upregulated in PGVG **C** or Phytol **D** exposed lung relative to control. Purple indicates metabolites downregulated in PGVG **C** or Phytol **D** relative to control. **C** Isobutyric acid and tryptophan represent the largest increase in PGVG while proline is largest downregulation versus control. **D** Phytol volcano plot with acetylcholine as the most upregulated in phytol versus FA while proline is most downregulated in phytol compared to FA control. For the full list of metabolites differentially detected in Phytol versus FA and PGVG versus FA, see Table 2

To dissect the effects of PGVG and PGVG + phytol exposures on both lung and systemic function, we performed lipidomic (Fig. 2) and metabolomic (Fig. 3) analyses on whole lung extracts. Based on a principal component analysis (PCA), PGVG + phytol-exposed animals did not significantly change lipid profile from FA control animals, while PGVG-exposed animals showed differences in both upregulated lung lipids (red, Fig. 2A) as well as downregulated lipids (blue, Fig. 2A). Using partial least squares discriminant analyses (PLS-DA) on the differentially expressed lipids in all 3 groups, we saw a large degree of overlap between FA and PGVG + phytol, while PGVG separated as a distinct group (Fig. 2B). Table 1 lists all the differentially upregulated and downregulated lipids between PGVG + phytol and FA (top of Table 1) versus PGVG and FA (bottom of Table 1).

**Table 1:** Statistically significantly different lipids for the comparisons of Phytol vs FA and PGVG vs FA

	Metabolite	FC	log2(FC)	−log10(p)
Phytol	Cer(d18:1/17:2)	0.00115	− 9.7704	1.5962
	Palmitic acid	0.03538	− 4.8211	1.4527
	PC 35:2	0.33585	− 1.5741	1.7379
	PE 35:5	2.523	1.3352	1.0466
	PE 38:4	3.6604	1.872	1.0291
	PC 32:2	4.3165	2.1099	1.782
	PG 32:0	4.4549	2.1554	1.1704
	PE 16-0,18-1-3	5.1563	2.3663	1.209
PGVG	Cer(d18:1/22:0)	0.00015	− 12.708	5.4973
	DG(18:2_18:0)	0.0002	− 12.298	4.1478
	PS 38:1	0.0002	− 12.266	5.4119
	Cer (d18:2/16:0)	0.0002	− 12.262	4.7184
	Cer(d18:2/24:1)	0.00021	− 12.251	4.1506
	LPE 18:0	0.00021	− 12.226	6.504
	DG(16:0_16:0)	0.00021	− 12.194	5.694
	Palmitic acid	0.00022	− 12.171	2.4323
	PC(18:0/18:0)	0.00022	− 12.121	4.5752
	PE 35:5	0.00023	− 12.095	3.3403
	Cer(d18:1/16:0)	0.00023	− 12.095	3.3403
	Cer(d18:1/25:0)	0.00023	− 12.079	4.2333
	Cardiolipin (18:1)(18:1)(18:1)(18:2)	0.00024	− 12.053	2.1101
	PS 34:1-1	0.00025	− 11.965	3.0063
	Cer(d18:1/24:1)	0.00026	− 11.926	4.045
	PE 35:3	0.00031	− 11.654	3.5414
	LPE 16:0	0.00033	− 11.579	5.3328
	PS 36:0-1	0.00035	− 11.475	3.5917
	PG 32:0	0.00042	− 11.232	3.7891
	PC 32:1	0.00052	− 10.91	2.6762
	PA(12:0/12:0)	0.00057	− 10.784	2.6539
	PC 36:4	0.00061	− 10.673	4.8443
	PC 32:2	0.00061	− 10.673	4.8443
	PC 37:7	0.00067	− 10.546	1.2964
	PI 38:4–1	0.00069	− 10.498	1.6448
	TG(18:1_18:1_18:1)	0.0007	− 10.477	1.6099
	DG(20:4_18:1)	0.00075	− 10.386	2.4363
	Stearic acid	0.00075	− 10.386	2.4363
	PE(18:0/18:1)	0.00083	− 10.236	2.5514
	LPC O-18:0	0.00097	− 10.015	2.3409
	Cer(d18:1/24:0)	0.0011	− 9.8221	2.1698
	DG(18:0_16:0)	0.00115	− 9.7704	2.504
	Cer(d18:2/18:0)	0.00123	− 9.6708	2.7196
	DG(20:4_16:0)	0.00125	− 9.6416	4.4494
	PC 38:6	0.00132	− 9.562	4.5345
	PI 36:4-1	0.00132	− 9.562	4.5345
	LysoPC (15:0/0:0)	0.00143	− 9.453	2.4432
	Cer(d18:2/24:0)	0.00144	− 9.4356	1.998
	PE 36:4	0.00157	− 9.3148	2.9661
	Cer(d18:1/17:2)	0.00179	− 9.1268	1.5558
	PC 36:0	0.00195	− 9.0015	1.4976
	PC 33:2	0.00211	− 8.8855	1.5757
	PC 38:5	0.00231	− 8.7603	5.5134
	Cer(d18:1/22:2)	0.00231	− 8.7603	5.5134
	PE 34:1	0.00239	− 8.7098	3.3126
	PG 34:2	0.00271	− 8.5277	3.3915
	PS 36:0-2	0.00424	− 7.883	2.4486
	PE 38:5	0.00888	− 6.8149	3.2615
	SM (d18:1/17:0)	0.0104	− 6.5869	3.5623
	PS 36:1-1	0.0148	− 6.078	2.6623
	PE 38:4	0.0287	− 5.1228	3.2156
	Lyso PC (18:1)	0.03076	− 5.0228	3.2135
	DG(18:1_16:1)	0.09277	− 3.4303	1.5821
	PC 34:0	0.24283	− 2.042	2.4338
	PS 40:6-1	0.38553	− 1.3751	1.3339
	PC(16:0/16:0)	2.6101	1.3841	1.4403
	PE 16-0,18-1-3	2.6101	1.3841	1.4403
	PC 36:1	3.1521	1.6563	1.2648
	PS 36:1-2	3.371	1.7532	1.3038
	PC 32:0	4.311	2.108	2.0607
	PC 35:2	4.311	2.108	2.0607
	DG 30:1	4.5135	2.1742	1.0087
	PS 38:4-2	5.075	2.3434	1.6867
	DG(18:1_18:0)	5.1499	2.3645	1.8566
	PI 34:1-1	6.7075	2.7458	1.5847
	PC 37:3	7.1003	2.8279	1.6506
	LPC 22:0	7.3551	2.8787	1.3532
	PS (12:0/12:0)	9.9955	3.3213	2.8123
	PG 34:1	13.834	3.7902	2.4479
	Cer(d18:1/22:1)	39.752	5.3129	3.2339
	Oleic acid	561.56	9.1333	2.1111
	PC 33:1	5005	12.289	4.8797
	PC 40:3	7228.6	12.819	4.4114
	Cardiolipin (18:1)(18:1)(18:1)(18:1)	7382.5	12.85	6.0367
	LPE 18:2	7860.1	12.94	6.6564
	PE (18:1/18:1)	8103.7	12.984	7.2583
	PE(18:0/18:0)	9072.4	13.147	7.6326
	Cer(d18:2/16:0)	9839.1	13.264	5.4926

Full list of all significantly different lipids between PGVG + phytol versus FA and also PGVG versus FA from Fig. 2

Based on individual lipid species/classes, PGVG exposure led to the upregulation of multiple Lysophosphatidylcholine (LysoPC) and Lysophosphatidylethanol (LysoPE) species compared to FA control (Fig. 2C, Table 1). Ceramides, Phosphatidylcholine (PC), and phosphatidylethanolamine species (PE) were significantly changed, with PE and PC species were upregulated in PGVG while ceramides were downregulated (Fig. 2C, Table 1). In contrast, a comparison of PGVG + phytol versus control revealed only a few lipids that were differentially regulated, with a specific ceramide (d18:1/17:2) downregulated in phytol compared to FA, along with palmitic acid. PC as well as several PE species were upregulated in phytol relative to FA (Fig. 2D, Table 1). Thus, exposure to PGVG and PGVG + phytol induced significantly different changes to the lung lipid environment compared to control.

### Lung metabolomics

We also determined the changes to metabolites in the lungs of animals after PGVG and Phytol exposures. In contrast to lipids, lung metabolites of PGVG-exposed mice showed similar patterns to FA controls, while PGVG + phytol-exposed mice showed significant differences, clustering as a separate group (Fig. 3A, B). Metabolites in lungs of PGVG-exposed animals showed significant changes from FA, with higher isobutyric acid, tryptophan, and AMP levels in PGVG compared to FA, while proline and TMAO were downregulated (Fig. 3C, for full list see Table 2). A select number of metabolites also varied between PGVG + phytol and FA (Table 2, top). The difference in metabolites between PGVG + phytol versus FA appeared greater than with PGVG versus FA (Table 2 bottom). Namely, acetylcholine and AMP were highly upregulated in PGVG + phytol-exposed lungs compared to FA, while proline and isobutyric acid were downregulated (Fig. 3D, Table 2). These results show that while PGVG and PGVG + phytol exposures both induced changes in metabolites and lipids compared to FA, but specific additives even at very low concentrations can significantly alter the biochemical responses to vaping exposure.

**Table 2:** Statistically significantly different metabolites for the comparisons of Phytol vs FA and PGVG vs FA

	Metabolite	FC	log2(FC)	−log10(p)
Phytol	Proline	0.002	− 8.9056	2.052
	Xylose	0.003	− 8.2901	1.1512
	Ribose	0.003	− 8.2664	1.1963
	Tyrosine	0.004	− 7.8646	1.0609
	Isobutyric acid	0.012	− 6.3739	1.2919
	Malate	0.151	− 2.7309	1.5298
	TMAO	0.255	− 1.9705	1.1509
	3-hydroxykynurenine	0.288	− 1.7961	1.0959
	AMP	201.1	7.6515	1.5679
	Acetylcholine	276.3	8.1101	1.9174
PGVG	Proline	0.003	− 8.3479	2.2478
	Leucine	0.005	− 7.785	1.4031
	Isoleucine	0.01	− 6.6981	1.2453
	Pyroglutamic acid	0.013	− 6.3186	1.0408
	TMAO	0.029	− 5.0939	2.3908
	PGE2	0.325	− 1.6206	1.2153
	4-Pyrodoxic acid	0.428	− 1.2253	1.0546
	Acetylcarnitine	2.198	1.1359	2.032
	3-Methylbutanoic acid	2.207	1.1418	2.2317
	Succinate	2.502	1.323	1.4882
	Methylmalonic acid	2.6	1.3782	1.5056
	Mannose	2.841	1.5063	1.5751
	Glucose	3.018	1.5934	1.6454
	L-Xylonate	3.774	1.916	2.0036
	AMP	21.49	4.4257	1.3367
	Isobutyric acid	27.44	4.7783	3.7767
	Tryptophan	482	8.9129	1.4201

Full list of all significantly different metabolites between PGVG + phytol versus FA and also PGVG versus FA from Fig. 3

### Lung transcriptomics

**Fig. 4:**
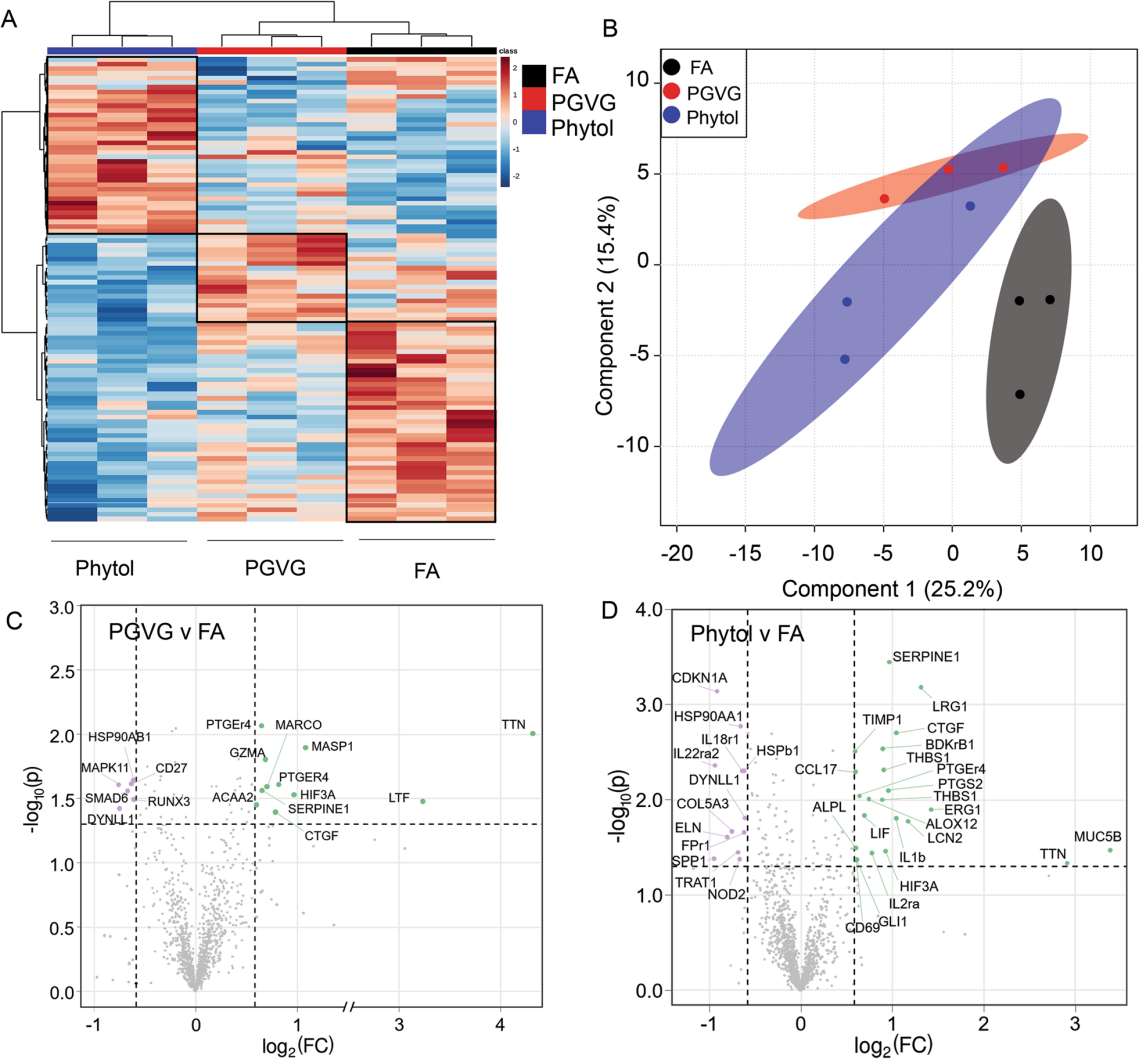
Alterations in mRNA transcripts in lungs following exposure to Ecig components PGVG and Phytol. Lungs from individual animals from each group (n = 3/gp) were frozen and RNA extracted for mRNA analysis using Fibrosis kit and Host Response kit using Nanostring technology. **A** Heat map comparing individual samples from the top 100 differentially expressed transcripts; **B** Overlay of statistically significant transcripts compiled from Host Response and Fibrosis kits using he partial least squares discriminant analysis (PLS-DA). **C**, **D** Volcano plot showing a comparison of statistically significant differentially expressed transcripts from lung of PGVG exposed **C** or Phytol exposed **D** versus filtered air control animals using results from both the Fibrosis and Host Response kits. Green indicates transcripts upregulated in PGVG **C** or Phytol **D** exposed lung relative to control. Purple indicates transcripts downregulated in PGVG **C** or Phytol **D** relative to control. For the full list of differentially expressed mRNA transcripts in Phytol versus FA and PGVG versus FA, see Table 3

To assess potential functional and inflammatory changes to the lung, we isolated RNA from lungs of PGVG-, PGVG + phytol-, and FA-exposed animals and analyzed gene expression via the Nanostring platform using both the Fibrosis and Host Response libraries. The RNA transcriptomic analysis showed that animals exposed to PGVG or PGVG + phytol separated cleanly from each other, and each separated from FA control by heat mapping (Fig. 4A), as well as PLS-DA analysis (Fig. 4B). PGVG exposure induced fewer transcriptional changes than PGVG + phytol but did induce upregulation of inflammatory genes granzyme A as well as *Ttn* (Table 3, bottom), and downregulation of *Runx3* and *Mapk11* (Fig. 4C, for full list see Table 3). Phytol exposure led to upregulation of multiple genes, including genes that regulate lung function such as *Muc5b*, *Serpine1*, and several inflammatory genes including *IL-1b* (Table 3 top). Phytol also induced downregulation of some inflammation related genes such as *Il-18r1* and *Il-22ra2* as well as extracellular matrix component *Col5a3* (Fig. 4D, Table 3). These results suggest that vaping exposure not only changes the overall lung environment but may modulate the immune compartment.

**Table 3:** Statistically significant differentially expressed transcripts for the comparisons of Phytol vs FA and PGVG vs FA

	RNA	FC	log2(FC)	Nanostring Adj P
Phytol	Spp1	− 1.93	− 0.95	0.04
	Il22ra2	− 1.9	− 0.93	0.00436
	Cdkn1a	− 1.89	− 0.92	0.000725
	Eln	− 1.75	− 0.81	0.02
	Col5a3	− 1.69	− 0.75	0.02
	Trat1	− 1.61	− 0.69	0.04
	Hsp90aa1	− 1.6	− 0.68	0.00229
	Nod2	− 1.58	− 0.66	0.04
	Hsp90aa1	− 1.58	− 0.66	0.00168
	Hspb1	− 1.55	− 0.63	0.00495
	Fpr1	− 1.53	− 0.61	0.02
	Il18r1	− 1.53	− 0.61	0.00495
	Dynll1	− 1.53	− 0.61	0.02
	Timp1	1.51	0.59	0.0031
	Alpl	1.52	0.6	0.03
	Ccl17	1.52	0.6	0.00509
	Gli1	1.52	0.61	0.04
	Cd69	1.53	0.62	0.04
	Ptger4	1.56	0.64	0.00911
	Lif	1.62	0.7	0.01
	Alox12	1.68	0.75	0.00983
	Il2ra	1.71	0.77	0.04
	Ptgs2	1.82	0.86	0.00752
	Thbs1	1.85	0.89	0.00999
	Thbs1	1.87	0.9	0.00487
	Bdkrb1	1.86	0.9	0.0029
	Hif3a	1.9	0.92	0.03
	Ptgs2	1.94	0.96	0.00801
	Serpine1	1.95	0.97	0.000359
	Il1b	2.06	1.04	0.02
	Ctgf	2.06	1.04	0.00199
	Lcn2	2.25	1.17	0.02
	Lrg1	2.48	1.31	0.000658
	Egr1	2.68	1.42	0.01
	Ttn	7.51	2.91	0.05
	Muc5b	10.4	3.38	0.03
PGVG	Cd27	− 1.55	− 0.63	0.02
	Runx3	− 1.52	− 0.61	0.03
	Mapk11	− 1.41	− 0.49	0.02
	Dynll1	− 1.4	− 0.48	0.04
	Smad6	− 1.32	− 0.4	0.03
	Hsp90ab1	− 1.27	− 0.34	0.02
	Acaa2	1.26	0.33	0.04
	Serpine1	1.31	0.39	0.03
	Gzma	1.34	0.42	0.02
	Marco	1.35	0.44	0.03
	Ctgf	1.43	0.52	0.04
	Ptger4	1.47	0.55	0.02
	Ptger4	1.57	0.65	0.00863
	Hif3a	1.95	0.96	0.03
	Masp1	2.11	1.08	0.01
	Ltf	9.51	3.25	0.03
	Ttn	19.79	4.31	0.00996

Full list of all significantly differentially expressed mRNA transcripts between PGVG + phytol versus FA and also PGVG versus FA from Fig. 4

### Alterations to Immune Cell Subsets in the Lung, Spleen, and Blood

**Fig. 5:**
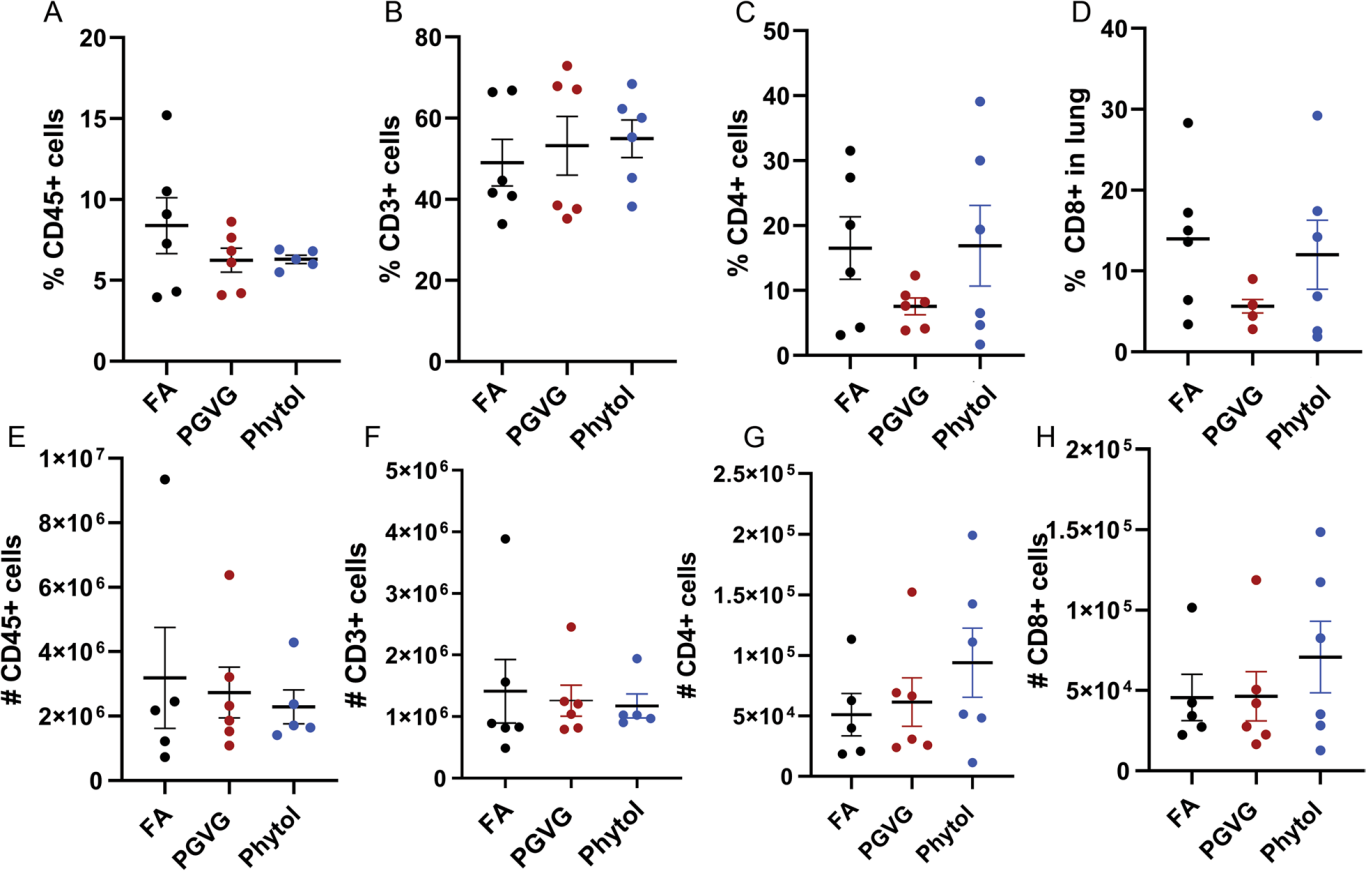
Vaping exposure does not significantly change T cell populations in lung. Animals were exposed to FA, PGVG or PGVG + phytol as described. At the end of the 8 week exposure, exposed animals were injected IV with anti-CD45 5 min prior to sacrifice, and lungs processed and stained and analyzed by flow cytometry for **A** extravascular % CD45 + , **B** CD3 + , **C** CD4 + , and **D** CD8 + cells, as well as **E** # of CD45 + , **F** CD3 + , **G** CD4 + , and **H** CD8 + cells. 2 independent experiments, n = 3 each for total of 6

To analyze potential changes to the cellular immune compartment after vaping, we analyzed immune cell subsets within the lung after exposure to PGVG as well as PGVG + phytol. After exposure as previously described, we analyzed cells from the interstitial lung tissue of exposed animals by injecting anti-CD45 antibody intravenously just prior to sacrifice. Cells labeled with IV-CD45 are in the vasculature while CD45-negative cells are in the tissue [29]. We found that exposure to PGVG and PGVG + phytol for 8 weeks did not significantly change total CD45 + (Fig. 5A, E), CD3 + (Fig. 5B, F), CD3 + CD4 + (Fig. 5C, G), or CD3 + CD8 + (Fig. 5D, H) cells within the lung either by percentage (Fig. 5A–D) or absolute number of cells (Fig. 5E–H). We also analyzed CD19 + , CD11b, and CD11c, and found no significant differences in the percentage or number of immune cells in the lung post vaping exposure (data not shown). These results agree with previous findings that vaping does not induce large scale changes to immune cell populations in the lung.

**Fig. 6:**
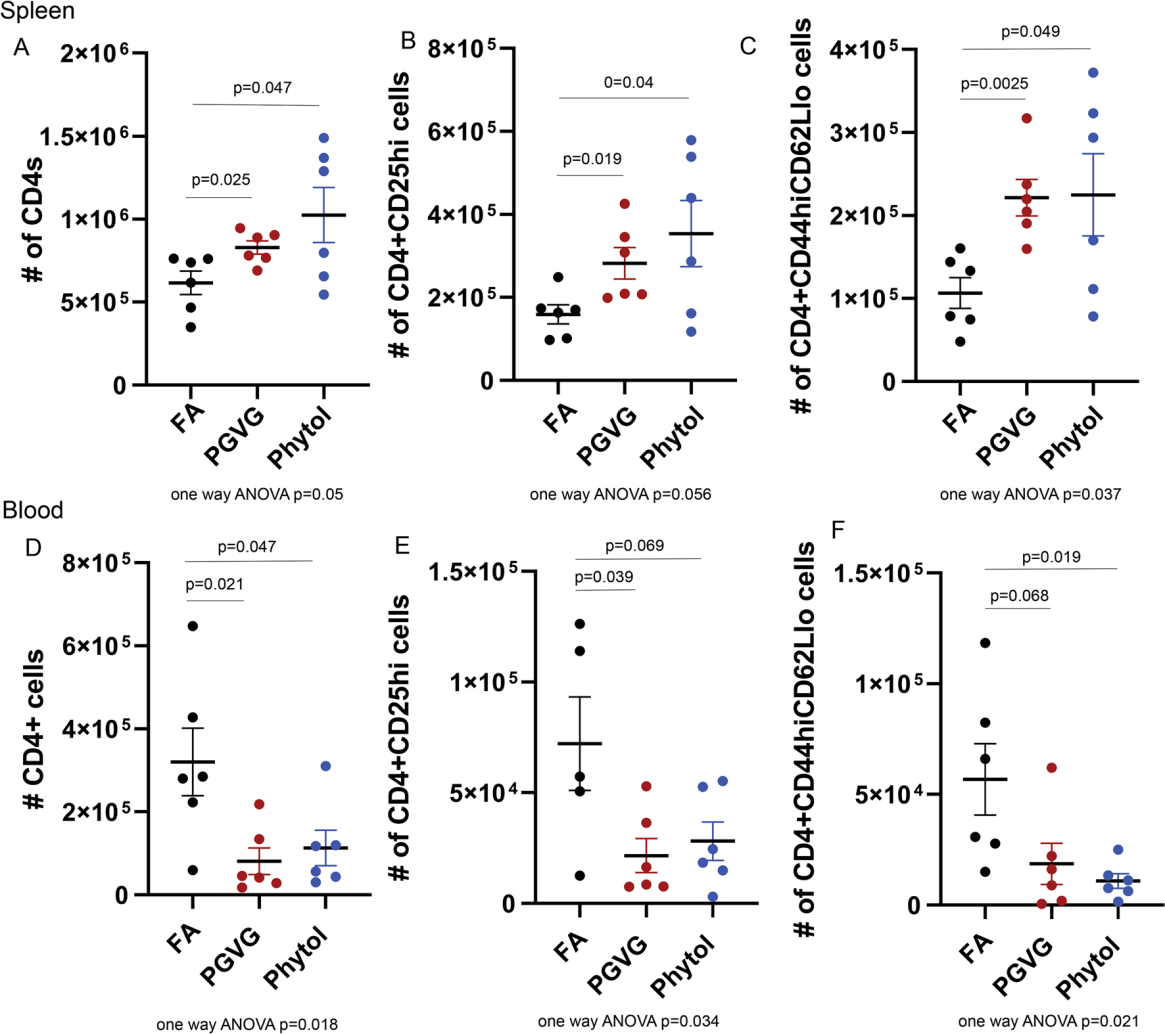
Vaping exposure significantly changes T cell populations in spleen and blood of exposed animals. Animals were exposed to FA, PGVG or PGVG + phytol as described. At the end of the 8 week exposure, exposed animals were injected IV with anti-CD45 5 min prior to sacrifice, and spleens **A**–**C** and blood **D**–**F** were processed, stained, and analyzed by flow cytometry for number of CD45 + CD3 + CD4 + T cells (**A**, **D**), CD45 + CD3 + CD4 + CD25hi T cells (B, E), and CD45 + CD3 + CD4 + CD44hiCD62Llo T cells (**C**, **F**). T-test p values between 2 conditions indicated by line 2 experiments, p value for one way ANOVA of all 3 conditions shown below. n = 3 each for total of 6

We also analyzed systemic and circulating immune cell subsets in animals exposed to PGVG and phytol. Interestingly, we found that both the spleen and blood showed significant changes to the CD4 compartment, particularly in the number of total CD4 T cells (Fig. 6A, D) as well as CD25hi (Fig. 6B, E) and CD44hiCD62Llo CD4 T cells (Fig. 6C, F) We found that PGVG and phytol exposures increased total CD4 (Fig. 6A), CD4 + CD25hi (Fig. 6B), and CD4 + CD44hiCD62Llo (Fig. 6C) in the spleen compared to FA control animals. In the blood, we saw a commensurate decrease in total CD4 (Fig. 6D), CD4 + CD25hi (Fig. 6E), and CD4 + CD44hiCD62Llo (Fig. 6F) in PGVG and phytol exposed animals compared to FA control. These results suggest that there may be an overall redistribution of CD4 T cells from blood to the spleen.

### Integration of multi-omic data

**Fig. 7:**
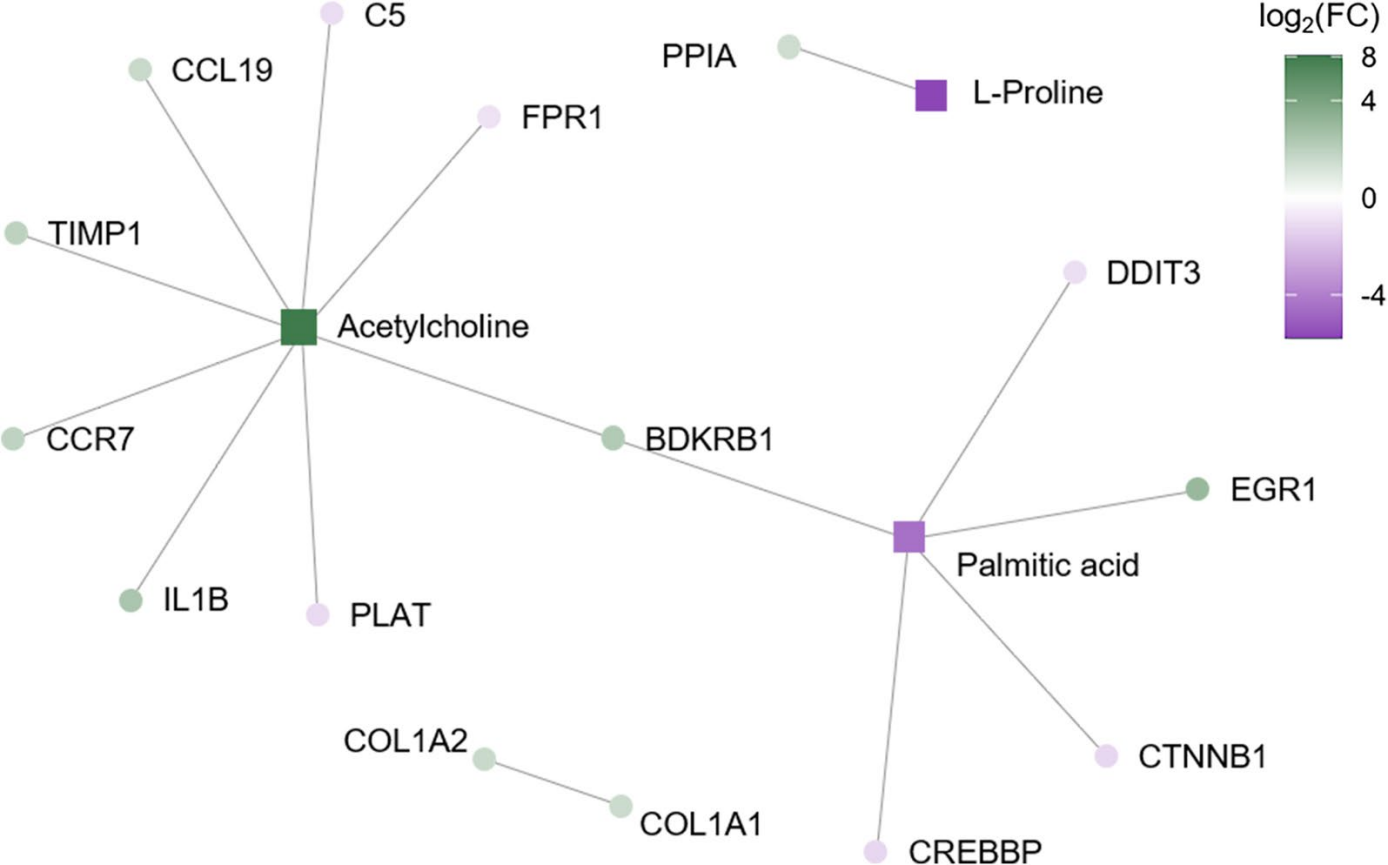
Multi-omic lung analysis combining transcriptome, lipidome, and metabolome comparing FA with Phytol. The resultant plot is based on the input of statistically significant molecules from Figs. 2, 3, 4. Each node represents a metabolite, lipid, or transcript, while lines represent linkers between nodes based on statistically significant pathways. For example, acetylcholine and bradykinin are both vasodilators and bronchoconstrictors. The acetylcholine metabolite and the bradykinin B1 receptor (BDKRB1) are linked, while BDKRB1 is linked to palmitic acid

We then assembled all the data together into an overall gene-metabolite interaction network for PGVG and PGVG + phytol exposure. This integrated statistical analysis revealed no overall network for the PGVG condition. Combining the data from the gene expression, metabolite, and lipid analyses, we find a few key nodes of upregulated and downregulated pathways upon PGVG + phytol exposure (Fig. 7). PGVG + phytol exposure led to excessive upregulation of acetylcholine, as well as key T cell chemokines, CCL19 and CCR7, which corroborates flow cytometry data showing changes to the T cell compartment in phytol exposed animals. PGVG + phytol exposure also led to downregulation of proline and palmitic acid. The key upregulated pathways (Table 4) and downregulated pathways (Table 5) in PGVG + phytol exposed animals are shown.

**Table 4:** Statistically significant upregulated reactome pathways after multi− omic analysis of PGVG + phytol lungs

Upregulated reactome	
Name	P-value
Crosslinking of collagen fibrils	2.47E−06
Platelet Adhesion to exposed collagen	2.96E−06
GPVI-mediated activation cascade	0.0000237
Degradation of the extracellular matrix	0.000131
Peptide ligand-binding receptors	0.000439
Chemokine receptors bind chemokines	0.00113
Class A/1 (Rhodopsin-like receptors)	0.00182
GPCR ligand binding	0.00414
Interleukin-1 processing	0.00449
Cytokine Signaling in Immune system	0.0245
GPCR downstream signaling	0.027
Activation of Matrix Metalloproteinases	0.0275
Signaling by GPCR	0.039
Interleukin-1 signaling	0.0398

**Table 5:** Statistically significant downregulated reactome pathways after multi-omic analysis of PGVG + phytol lungs

Downregulated reactome	
Name	P-value
Activation of Chaperone Genes by ATF6-alpha	0.00449
Signal Transduction	0.00487
Formyl peptide receptors bind formyl peptides and many other ligands	0.00538
Activation of C3 and C5	0.00628
Terminal pathway of complement	0.00628
Activation of Genes by ATF4	0.00628
Activation of Chaperones by ATF6-alpha	0.00717
Regulation of Gene Expression by Hypoxia-inducible Factor	0.00806
Dissolution of Fibrin Clot	0.00896
Peptide ligand-binding receptors	0.0114
Apoptotic cleavage of cell adhesion proteins	0.0116
PERK regulated gene expression	0.0116
TRAF3-dependent IRF activation pathway	0.0125
NICD traffics to nucleus	0.0134
Beta-catenin phosphorylation cascade	0.0134
Notch-HLH transcription pathway	0.0134
Cellular responses to stress	0.024
Cellular response to hypoxia	0.024
Regulation of Hypoxia-inducible Factor (HIF) by Oxygen	0.024
TRAF6 mediated IRF7 activation	0.0249
Myogenesis	0.0275
CDO in myogenesis	0.0275
Adherens junctions interactions	0.0275
Class A/1 (Rhodopsin-like receptors)	0.0288
Complement cascade	0.0293
Apoptotic cleavage of cellular proteins	0.038
NOTCH1 Intracellular Domain Regulates Transcription	0.0441
Constitutive Signaling by NOTCH1 HD + PEST Domain Mutants	0.0458
GPCR ligand binding	0.0489
Transcriptional Regulation of White Adipocyte Differentiation	0.0493
Developmental Biology	0.0493

## Discussion

As Ecig use has become more widespread, the term “epidemic” has been applied to the ubiquitous use of Ecigs. Many users switch from nicotine products to Ecigs believing vaping to be a safer alternative, but little is actually known about the effects of vaping on physiological processes. The United Kingdom has largely deemed vaping as a safer alternative to conventional smoking. However, vaping is difficult to study due to the large number of sometimes unknown additives that are contained within unregulated vaping products.

The recent outbreak of vaping-associated acute lung injury (EVALI), traced to the additive Vitamin E acetate, demonstrated the potential harm posed by vaping components. In this study, we focused on the effects of exposure to the base fluid contained in many vaping systems, PGVG, as well as a novel terpene additive, phytol found in counterfeit cartridges [24]. We find that PGVG and PGVG + 1% phytol elicits both lung-specific and systemic effects on multiple systems, including metabolites, lipids, and specific subsets of immune cells. Our results suggest that PGVG with and without phytol altered lipidomic, metabolomic, and transcriptomic profiles of multiple systems and even modified immune cell populations outside the lung. These results agree well with previous published studies showing that PGVG alone can alter lung function, lipid composition and T cell subsets [27, 28]. However, when put into the context of systemic and multi-omic pathway integration, the effects of PGVG alone are quite modest. Namely, PGVG alone did not produce a statistically significant multi-omic pathway.

Additives or alternative ingredients—such as phytol added to the base liquid PGVG in vaping products—can combine for unpredictable effects, as was the case for vitamin E acetate [22, 23]. The addition of phytol modified the lung-omic profiles relative to PGVG alone, but several common outcomes were noted. Both PGVG and PGVG + 1% phytol upregulated AMP and downregulated proline, although they appear to have opposing effects on isobutyric acid, with PGVG increasing isobutyric acid levels while PGVG + phytol decreased levels. Changes in gene transcription and metabolites were quite different between PGVG alone and PGVG + phytol, and the different effects on weight gain between PGVG and PGVG + phytol are likely to result from systemic metabolic and transcriptomic effects. Connective tissue growth factor (CTGF), known for its involvement in wound healing and pathological lung remodeling [30, 31], was elevated in both exposure groups. On the other hand, 1% phytol led to a significant upregulation of MUC5B, a regulator of mucin production in the airways important for mucociliary clearance and associated with fibrosis in the lung [32, 33]. In contrast, while we found PGVG did not affect MUC5B, a previous study showed increased Muc5ac in lungs of vaping exposed animals [28], suggesting that additives and exposure conditions may affect similar lung repair pathways. PGVG did significantly change multiple lipid species including many ceramides, Phosphatidylcholine (PCs), and phosphatidylethanolamine species (PEs), while 1% Phytol + PGVG exposure evinced only modest alteration of lipids. Ceramide increases have been associated with lung injury, particularly COPD [34, 35], while PEs and PCs are components of lung surfactant, critical for alveolar expansion for gas exchange [34–38]. Lipids have been found to be changed in the plasma of a small group of Ecig users, including similar lipid species to those identified in our studies [39]. These results point to a complex interplay and unpredictable interactive effects between additives such as phytol with base liquids. Complex addition of nicotine, cannabinoids, flavorings, etc., has the potential to affect multiple physiological systems, including metabolic and immune systems, in a manner that is currently difficult to predict.

Little is known about the novel diterpene alcohol phytol found in Ecig products [24]. An extremely high dose (100%) of phytol was shown to be lethal to rats [25], but in our studies we find that even at a very low dose, 1%, phytol can induce significant changes to lung transcription, metabolites, and even function. Using analysis combining our results showing changes in transcriptomics, lipidomics, and metabolomics, we find that phytol increases acetylcholine. Acetylcholine is a potent endogenous bronchoconstrictive agent [40–43]. However, we find that 1% phytol possibly decreases resistance (non-significant trend) and increases lung compliance. Given the extent of lipidomic changes seen in the present study and elsewhere [9], increased acetylcholine could reflect compensatory mechanisms in vaping-exposed lungs to offset physicochemical changes in the airway surfactant. Furthermore, the upregulation of bradykinin receptor B1 could indicate a request for additional vasodilation and/or bronchoconstriction. Through the linkage to a downregulated palmitic acid, the effects observed could partially describe an inappropriate response to pathogens during sterile conditions, and an increased vulnerability to pathogen insult.

We also show phytol had significant effects on immune genes, including regulators of immune cell migration (CCL19, CCR7), inflammation (IL-1β), and immune regulation (TIMP1). Both PGVG and PGVG + phytol changed the circulating CD4 + T cell population, with decreased numbers in the blood and increased numbers in the spleen, both in total CD4s as well as CD44hi and CD25hi populations. Changes to the CD4 T cell subset were also previously observed in animals exposed to VEA in an EVALI model, suggesting that Ecig exposure may broadly affect the CD4 T cell response [44]. Interestingly, while we find no changes in overall immune subsets in the lung, one previous study did show some change in CD19 + and CD4 + cells in the lung [27]. These results suggest that changes to metabolites, lipids, and gene products in the lung may translate into systemic changes, including systemic changes to immune cell subsets. Such alterations to the systemic T cell population may only manifest under the circumstance of a lung immunological challenge (*i.e.*, a respiratory pathogen). Epidemiological data have been reported showing individuals who vape exhibit increased susceptibility to infectious disease [6, 45]. Our results provide a potential mechanism tying exposures in the lung to systemic changes in immunity.

These results must be put into context with the operating conditions, which included a specific Ecig device, coil, and wattage. Sub-ohm vaping is not an uncommon practice among users but is more prone to high temperatures at the coil, which can generate numerous pyrolysis products [46, 47]. We endeavored to keep the puff topography (short puffs, longer intervals between puffs) to avoid overheating conditions, however, we did not explicitly measure products like metals, acrolein, or formaldehyde that could have been generated. Furthermore, additives including triacetin can be found in Ecig liquids, particularly in devices that contain THC or cannabis [48, 49], which can also lead to formation of acrolein and formaldehyde upon vaping [50]. Our addition of phytol was a simple individual study permutation that clearly altered the response phenotype. We predict that numerous other ingredients and aerosol generating conditions can also change the responses.

Changes to lipids, metabolites, and genes can lead to a multitude of downstream effects, but in the present study we have limited phenotyping showing modest alterations of lung function. Lung disease is often multifactorial, with even pathologies from long-term cigarette smoking being heavily influenced by genetics and lifestyle. The mulit-omic alterations observed in response to PGVGV and PGVG + phytol may simply amount to moderate homeostatic adjustments to an altered chemistry of the airway lining fluid. The data are concerning, however, with respect to the complex differences with a small amount of terpene added to the base, and in consideration of systemic immune cell population changes. Long-term Ecig use overlaid with a vulnerable genetic profile or in the face of a second hit (e.g., pathogens or toxicants), may lead to more pathological alterations to respiratory health. While these remain to be determined, our study demonstrates the importance of testing the effects of specific compounds in combination due to the complex interplay of toxicological exposures with biological processes.

## Materials and methods

### Mice and reagents

C57BL/6 from Jackson Laboratories were used. All mice were maintained in a specific pathogen-free environment in barrier facilities at the University of New Mexico School of Medicine in Albuquerque, NM, and conform to the principles outlined by the Animal Welfare Act and the National Institutes of Health guidelines and approved by the IACUC animal use committee. Females were used at between 8–20 weeks. All experimental protocols were approved by the IACUC animal use committee at UNM Health Sciences Center in accordance with relevant guidelines and regulations (IACUC protocol #s: 18-200797-B-HSC; 16-200497-HSC; 19-200892-HSC). All animal work was performed and reported according to ARRIVE guidelines.

### Reagents used

Antibodies used include: anti-mouse CD45 APCCY7 (Clone 30-F11 Biolegend Cat #103116) and anti-mouse CD45 Eflour 450 (Clone 30-F11 Invitrogen Cat#48-0451-82); anti-mouse LY6G (Clone 1A8; Biolegend Cat#127623); anti-Mouse CD3 Pacific Blue (Clone 145-2C11 Biolegend Cat#100334); anti-Mouse CD3e, PerCP-Cy5.5 (Clone: 145-2C11, eBioscience, Cat#: 45-0031-82); anti-Mouse CD8a PerCP (Clone Ly-2 BDBiosciences Cat#M037858); anti-Mouse CD4, FITC (Clone: GK1.5; eBioscience, Cat#: 25-0041-82); anti-mouse CD19 PE (Clone eBio1D3 ThermoFisher Cat# 12-0193-83); anti-mouse CD11c APC (Clone N418; Biolegen Cat #117310); anti-mouse CD11b FITC (Clone M1/70.15 Invitrogen Cat #RM2801); anti-mouse CD25 PE (Clone 3C7 Biolegend Cat#101904); anti-mouse CD44 PE (Clone 1M7; Biolegend Cat# 10,008); anti-mouse CD62L APC (Clone Mel-14 Biolegend Cat# 104411).

### Exposure

We developed a customized, flexible exposure system built on the InExpose (Scireq, Inc) cigarette exposure platform. Our modular, adjustable system allowed for a variety of commercially-available devices and e-liquids to be incorporated into our study design. The InExpose software/hardware system permitted real-time control of air flow from the vape device through the whole-body exposure chamber. A linear actuator was connected to adjustable timers to alter the duration and intervals of vape puffs. We used a Smok® G-Priv3 Mod, which allowed for adjustment of wattage up to 230W (50W with a 0.17Ω nickel coil was used for all data), used rechargeable batteries, provided a warning when resistance (ohms) or temperatures were out of range, and flexible use of a variety of commercial or laboratory-prepared e-liquids. Our platform was easily modified to incorporate other commercial vape devices and, naturally, accommodate conventional cigarettes. The exposure chamber was monitored in real-time for mass concentration (DustTrak II, TSI), targeting an average of 125 mg/m^3^ (Additional file 1: Fig. S1), based largely off of prior work measuring urine cotinine levels in exposed mice (Irfan 200 mg/m^3^: [51, 52]). The system was controlled as a stable concentration from day to day, despite the intermittent nature of the “puff” settings. The frequency and duration of puffs ranged from 2–3 per minute and 2–5 s per puff; these were manually varied throughout the exposure period to ensure comparable overall mass concentration levels. Dilution air was pulled through the exposure chamber (4.3L volume) at a manually adjusted rate of 1–3 lpm. A small fan was mounted in the exposure chamber to enhance mixing and consistency of exposures between mice. Aerosol size distribution (mmad typically 120-250 nm) was also characterized for different e-liquids of device/settings using a Laser Aerosol Spectrometer (TSI). The exposure chamber housed 16 mice. For safety, an O_2_-capnograph continuously monitored oxygen and carbon dioxide levels in exposure chambers.

C57BL/6 mice were exposed by inhalation for 2 h/day, 5 days/week × 8 weeks to a vaporized mixture of propylene glycol/vegetable glycerin (PGVG) (50% + 50%) with and without 1% phytol by volume.

### Pulmonary function

Post-exposure, a subset of mice from each group were anaesthetized via isoflurane and tracheostomized with a 19-gauge cannula before pulmonary function measurement using a Flexivent system (SCIREQ Scientific Respiratory Equipment), as previously described [53]. Resistance and compliance measurements were recorded in multiple intervals over a 5-min period. Methacholine challenge was omitted in these studies as we sought to capture baseline changes in respiratory function due to the exposure conditions. Data was compiled using FLEXIWARE software, version 7.6, and statistical analysis was performed in Graphpad Prism8.

### Transcriptomic analysis

Following sacrifice, lungs were extracted and placed in ice-cold PBS without calcium or magnesium until batch dissociation could be performed. The Miltenyi Biotec Lung Dissociation Kit (cat. No. 130-095-927) was employed, and the associated protocol followed, as it has been developed to minimize cell death during the single cell suspension steps on the gentleMACS™ Dissociators. One lung lobe per mouse was placed in a gentleMACS C Tube containing an enzymatic digestion solution, and manually cut into sections < 5 mm. Lungs were initially dissociated on the gentleMACS Octo Dissociator under heated conditions using the 37C_m_LDK_1 program. Tubes were rotated for 30 min at 37 °C before reattaching to the Octo Dissociator and running the m_lung_02 program. Sample suspensions were strained (70um pores) to remove large debris, and strainers were washed with a kit buffer. Subsequently, tubes were centrifuged at 300 × g for 10 min to pellet cells and supernatant was aspirated. Cells were resuspended and RNA was extracted using a commercial kit.

Lung Fibrosis and Host Response kits were purchased from Nanostring based on their targeted primer approach. Data was analyzed by ROSALIND® (https://rosalind.bio/), with a HyperScale architecture developed by ROSALIND, Inc. (San Diego, CA). Read Distribution percentages, violin plots, identity heatmaps, and sample MDS plots were generated as part of the QC step. Normalization, fold changes and p-values were calculated using criteria provided by Nanostring. ROSALIND® follows the nCounter® Advanced Analysis protocol of dividing counts within a lane by the geometric mean of the normalizer probes from the same lane. Housekeeping probes to be used for normalization are selected based on the geNorm algorithm as implemented in the NormqPCR R library1. Abundance of various cell populations is calculated on ROSALIND using the Nanostring Cell Type Profiling Module. ROSALIND performs a filtering of Cell Type Profiling results to include results that have scores with a p-Value greater than or equal to 0.05. Fold changes and pValues are calculated using the fast method as described in the nCounter® Advanced Analysis 2.0 User Manual. P-value adjustment is performed using the Benjamini–Hochberg method of estimating false discovery rates (FDR). Clustering of genes for the final heatmap of differentially expressed genes was done using the PAM (Partitioning Around Medoids) method using the fpc R library2 that takes into consideration the direction and type of all signals on a pathway, the position, role and type of every gene, etc. Hypergeometric distribution was used to analyze the enrichment of pathways, gene ontology, domain structure, and other ontologies. The topGO R library3, was used to determine local similarities and dependencies between GO terms in order to perform Elim pruning correction. Several database sources were referenced for enrichment analysis, including Interpro4, NCBI5, MSigDB6,7, REACTOME8, WikiPathways9. Enrichment was calculated relative to a set of background genes relevant for the experiment.

### Metabolomic and Lipidomic analysis

For metabolite extraction, each tissue sample (~ 20 mg) was homogenized in 200 µL MeOH:PBS (4:1, v:v, containing 1,810.5 μM ^13^C_3_-lactate and 142 μM ^13^C_5_-glutamic Acid) in an Eppendorf tube using a Bullet Blender homogenizer (Next Advance, Averill Park, NY). Then 800 µL MeOH:PBS (4:1, v:v, containing 1,810.5 μM ^13^C_3_-lactate and 142 μM ^13^C_5_-glutamic Acid) was added, and after vortexing for 10 s, the samples were stored at -20 °C for 30 min. The samples were then sonicated in an ice bath for 30 min. The samples were centrifuged at 14,000 RPM for 10 min (4 °C), and 800 µL supernatant was transferred to a new Eppendorf tube. The samples were then dried under vacuum using a CentriVap Concentrator (Labconco, Fort Scott, KS). Prior to MS analysis, the obtained residue was reconstituted in 150 μL 40% PBS/60% ACN. A quality control (QC) sample was pooled from all the study samples.

For lipid extraction, each tissue sample (~ 20 mg) was mixed with 200 µL 10x-diluted PBS (4 °C) and 80 µL internal standard solution (PC (17:0/17:0) and PG (17:0/17:0) in MeOH; 50 uM; 4 °C) in an Eppendorf tube (1.5 ml). Then stainless beads were added into the tube, and homogenization (2 min) was performed using a Bullet Blender homogenizer (Next Advance, Averill Park, NY). After homogenization, 400 µL MTBE were added into the sample. Then the sample was vortexed for 30 s, stored under − 20 °C for 30 min, and sonicated in an ice bath for 10 min. After centrifugation (14,000 rpm, 10 min), 300 µL upper MTBE layer was collected into a new Eppendorf tube. The MTBE layer was then dried in a Vacufuge Plus Evaporator. Samples were then reconstituted with 100 μL 1:1 IPA:MeOH. 80 μL of each sample was transferred to a LC-MS vial for analysis, while the remaining 20 μL was pooled to create a quality control (QC) sample. The QC was measured every 10 samples to ensure consistent output by the LC–MS/MS.

The untargeted LC-MS metabolomics method used here was modeled after that developed and used in a growing number of studies [54–57]. Briefly, all LC-MS experiments were performed on a Thermo Vanquish UPLC-Exploris 240 Orbitrap MS instrument (Waltham, MA). Each sample was injected twice, 1 µL for analysis using negative ionization mode and 1 µL for analysis using positive ionization mode. Both chromatographic separations were performed in hydrophilic interaction chromatography (HILIC) mode on a Waters XBridge BEH Amide column (150 × 2.1 mm, 2.5 µm particle size, Waters Corporation, Milford, MA). The flow rate was 0.3 mL/min, auto-sampler temperature was kept at 4 °C, and the column compartment was set at 40 °C. The mobile phase was composed of Solvents A (10 mM ammonium acetate, 10 mM ammonium hydroxide in 95% H_2_O/5% ACN) and B (10 mM ammonium acetate, 10 mM ammonium hydroxide in 95% ACN/5% H_2_O). After the initial 1 min isocratic elution of 90% B, the percentage of Solvent B decreased to 40% at t = 11 min. The composition of Solvent B maintained at 40% for 4 min (t = 15 min), and then the percentage of B gradually went back to 90%, to prepare for the next injection. Using mass spectrometer equipped with an electrospray ionization (ESI) source, we will collect untargeted data from 70 to 1050 m/z.

The untargeted LC–MS lipidomics method used here was modeled after that developed and used in a growing number of studies [58–60]. All mass spectrometry experiments were done on a Thermo Vanquish UPLC-Exploris 240 Orbitrap MS system (Waltham, MA). Each sample was run twice; one for positive ion mode and one for negative ion mode. For positive mode, 1 μL was used per injection, whereas 1 μL was used in negative ion mode injections. Both modes used reverse phase chromatography with a Waters XSelect HSS T3 column (150 × 2.1 mm, 2.5 µm particle size; Waters Corporation, Milford, MA). Flow through the column was maintained at 0.3 mL/min. The mobile phase Solvent A was comprised of 10 mM ammonium acetate in 60% H_2_O/40% ACN. Solvent B consisted of 10 mM ammonium acetate in 90% IPA/10% ACN. An isocratic elution was used with 50% solvent B for 3 min before its percentage was gradually increased to 100% over the next 12 min. Following 10 min of continued 100% solvent B, at t = 25 min, the percent of B was decreased gradually back to 50% to prepare for the next sample injection. Using mass spectrometer equipped with an electrospray ionization (ESI) source, we will collect untargeted data from 100 to 2000 m/z.

To identify peaks from the MS spectra, we made extensive use of the in-house chemical standards (~ 600 aqueous metabolites and (~ 800 lipids), and in addition, we searched the resulting MS spectra against the HMDB library, Lipidmap database, METLIN database, as well as commercial databases including mzCloud, Metabolika, and ChemSpider. The absolute intensity threshold for the MS data extraction was 1000, and the mass accuracy limit was set to 5 ppm. Identifications and annotations used available data for retention time (RT), exact mass (MS), MS/MS fragmentation pattern, and isotopic pattern. We used the Thermo Compound Discoverer 3.3 software and LipidSearch 4.2 software for aqueous metabolomics and lipidomics data processing, respectively. The untargeted data were processed by the software for peak picking, alignment, and normalization. To improve rigor, only the signals/peaks with CV < 20% across quality control (QC) pools, and the signals showing up in > 80% of all the samples were included for further analysis.

Resultant metabolomic and lipidomic datasets were input into metaboanalyst.ca. Batch runs contained QC samples at intervals of 1 QC per every 5 experimental samples and were used for initial normalization. Sample-wise, normalized by sum to make samples more comparable to each other. Featurewise, log transformed to correct for heteroscedasticity, and mean centered to focus on the differences rather than the similarities of the data. Heatmaps were autoscaled by features, with Euclidean distance, and Ward clustering. Top number of metabolites/lipids/RNA are described per figure legend. PLS-DA components were generated by DiscrMiner (v. 0.1–29) and plotted with a 95% confidence region ellipse. Volcano plots were generated by MeataboAnalystR (v. 2.0.0) for metabolites and lipids by FDR p < 0.1, minimum fold-change of 1.5.

### Transcriptomic, metabolomic, and lipidomic integration

Statistically significant RNA, metabolites, and lipids were compiled into lists and converted from mouse to human using the Rstudio convertid package (v. 0.1.3) based on the requirements of the MetaboAnalystR package. Knowledge-based network generation utilized each input value as seeds to map networks and subnetworks with neighbors. Our parameters (degree min = 2, betweenness min = 1) resulted in a single first-order subnetwork continent when examining metabolite/lipid-gene interactions and 11 subnetworks when examining metabolite/lipid-gene-disease interaction networks. The top subnetwork for each examination was compiled and pruned. I.e. subnetwork 1 from the metabolite/lipid-gene-disease interaction network contained 52 nodes, 54 edges, and 21 seeds, but 2 islands are presented here. The entirety of the metabolite/lipid-gene network is presented. Pruning was based on disease states that could not exist within the lungs, such as Alzheimer’s and schizophrenia connections with PC(16:0/18:2(9Z,12Z)) and acetylcholine, respectively.

### Flow cytometry

Single cell suspensions from murine LNs and spleens were stained with conjugated antibodies (listed in Mice and reagents) according to standard protocols. Data was acquired using a BD LSR Fortessa (BD) and analyzed using FlowJo (FlowJo, LLC). Flow cytometric analysis of primary murine cells consisted of gating on lymphocytes population from FSC x SSC, then on the indicated markers as shown.

## Supplementary Information

**Additional file 1: Figure S1. **Exposure Chamber and Vapor Concentration Quantification. **A** Ecig exposure setup, red arrows indicate direction of vapor flow. The linear actuatoris wired to an automatic relay timer which allows for modulation of vape duration and time between vape durations. The ecig moduleis affixed in place by removable screws. A black tube inserted into to the mouthpiece. This tube narrows into a blue tube, which leads to the vapor chamber. The vapor is sucked from the chamber by a pumpwhose flow rate can be altered via software. The pump exit tube leads to the mouse exposure chamber. An internal, isolated circulation fan is connected to an external power source. Underneath the exposure chamber is an exit tube that leads to the DustTrak particulate monitoring device, which allows for the quantification of particles leaving the chamber. **B** Representative quantification of a single 2-h exposure. Dots: vapor quantification every 1 min. The sinusoidal pattern is representative of linear actuator striking, followed by the time between strikes. **C** Average concentrations per day over a 31-day period. Dots: Average for each day. **C** Final average concentration over 31 days of exposure. **B**, **C** Grey: Loess local polynomial regression per condition. **D** Total averaged concentration over 31-day exposure.

**Additional file 2: Figure S2. **Cardiac function after Ecig exposure. Female mice ages 8–20 weeks were exposed to filtered air, propylene glycol/vegetable glycerinor PGVG + 1% phytol. Exposures occurred for 8 weeks, 2 h per day, 5 days per week. Mice were analyzed for cardiac function including **A** heart rate; **B** election fraction; **C** stroke volume; **D** diastolic dimension; **E** 1st derivative of the A wave, and **F** Function of the left ventricle represented by 1st derivative E wave/1st derivative A wave.

## Data Availability

All data are available upon request to the corresponding author including transcriptomic, lipidomic, and metabolomic data as well as analysis.
