# Supplementary figures and images for: Multi-omic assessment shows dysregulation of pulmonary and systemic immunity to e-cigarette exposure
Source: Respir Res. 2023 May 25;24:138. doi: 10.1186/s12931-023-02441-2 (PMC10209577; doi:10.1186/s12931-023-02441-2)

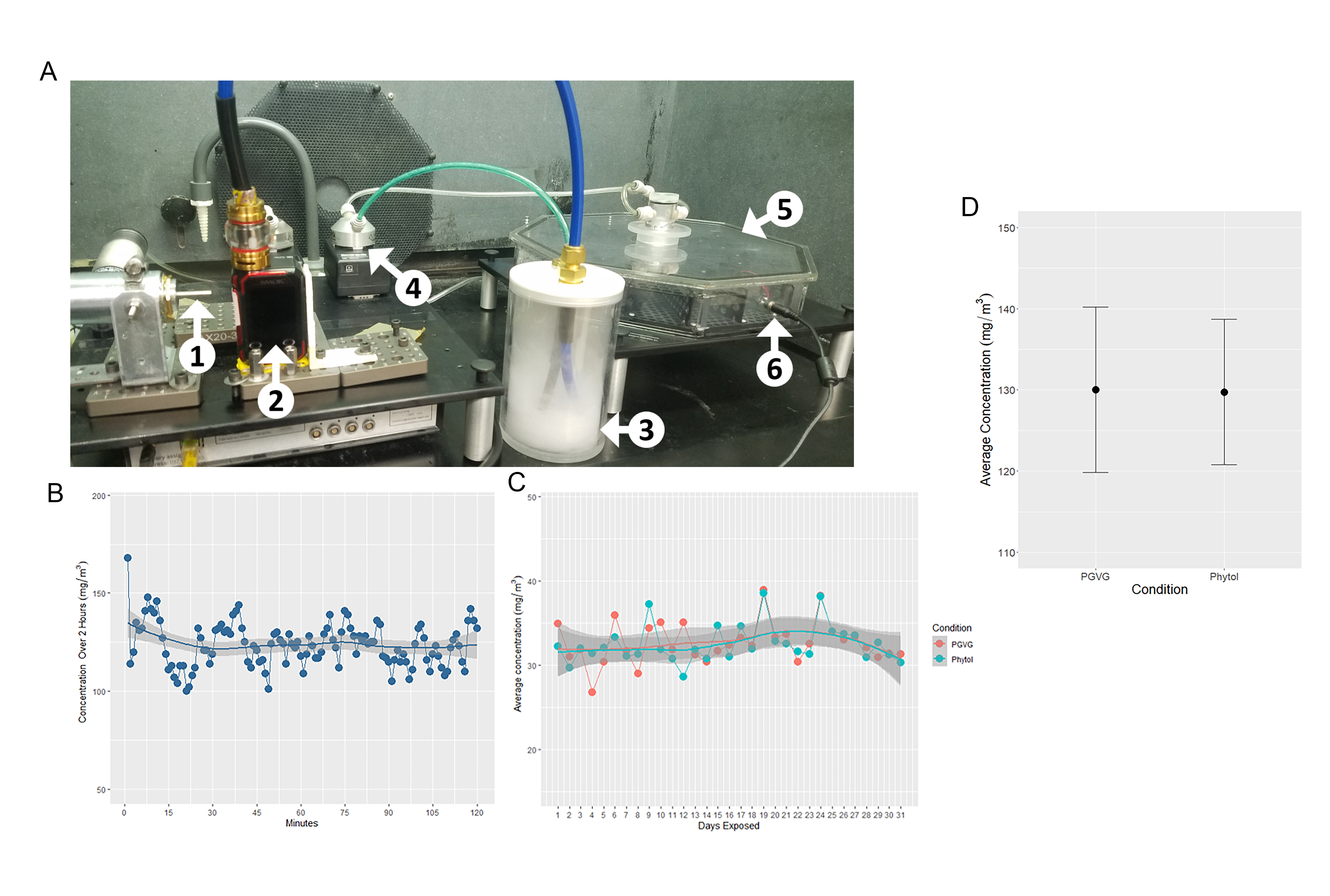

Supplement: Supplementary file 1 — Additional file 1: Figure S1. Exposure Chamber and Vapor Concentration Quantification. A Ecig exposure setup, red arrows indicate direction of vapor flow. The linear actuatoris wired to an automatic relay timer which allows for modulation of vape duration and time between vape durations. The ecig moduleis affixed in place by removable screws. A black tube inserted into to the mouthpiece. This tube narrows into a blue tube, which leads to the vapor chamber. The vapor is sucked from the chamber by a pumpwhose flow rate can be altered via software. The pump exit tube leads to the mouse exposure chamber. An internal, isolated circulation fan is connected to an external power source. Underneath the exposure chamber is an exit tube that leads to the DustTrak particulate monitoring device, which allows for the quantification of particles leaving the chamber. B Representative quantification of a single 2-h exposure. Dots: vapor quantification every 1 min. The sinusoidal pattern is representative of linear actuator striking, followed by the time between strikes. C Average concentrations per day over a 31-day period. Dots: Average for each day. C Final average concentration over 31 days of exposure. B, C Grey: Loess local polynomial regression per condition. D Total averaged concentration over 31-day exposure. [file 12931_2023_2441_MOESM1_ESM.tif]

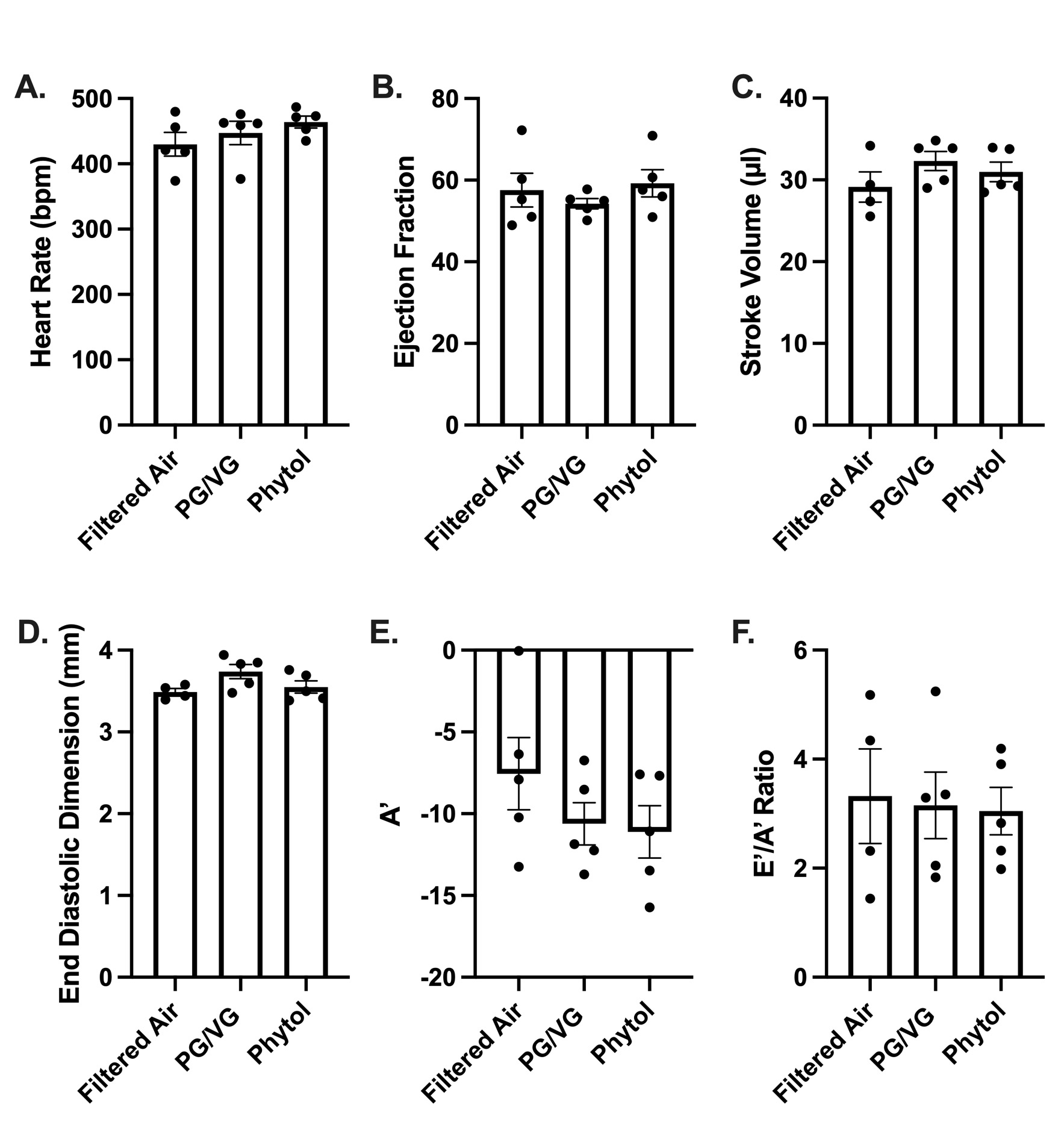

Supplement: Supplementary file 2 — Additional file 2: Figure S2. Cardiac function after Ecig exposure. Female mice ages 8–20 weeks were exposed to filtered air, propylene glycol/vegetable glycerinor PGVG + 1% phytol. Exposures occurred for 8 weeks, 2 h per day, 5 days per week. Mice were analyzed for cardiac function including A heart rate; B election fraction; C stroke volume; D diastolic dimension; E 1st derivative of the A wave, and F Function of the left ventricle represented by 1st derivative E wave/1st derivative A wave. [file 12931_2023_2441_MOESM2_ESM.tif]
